# Enhancing Brain Retention of a KIF11 Inhibitor Significantly Improves its Efficacy in a Mouse Model of Glioblastoma

**DOI:** 10.1038/s41598-020-63494-7

**Published:** 2020-04-16

**Authors:** Gautham Gampa, Rajappa S. Kenchappa, Afroz S. Mohammad, Karen E. Parrish, Minjee Kim, James F. Crish, Amanda Luu, Rita West, Alfredo Quinones Hinojosa, Jann N. Sarkaria, Steven S. Rosenfeld, William F. Elmquist

**Affiliations:** 1 Brain Barriers Research Center, Department of Pharmaceutics, College of Pharmacy, University of Minnesota, Minneapolis, MN USA; 2 Department of Cancer Biology, Mayo Clinic, Jacksonville, FL USA; 3 Department of Cancer Biology, Cleveland Clinic, Cleveland, OH USA; 4 Department of Neurologic Surgery, Mayo Clinic, Jacksonville, FL USA; 5 Department of Radiation Oncology, Mayo Clinic, Rochester, MN USA

**Keywords:** Cancer, Drug discovery, Oncology

## Abstract

Glioblastoma, the most lethal primary brain cancer, is extremely proliferative and invasive. Tumor cells at tumor/brain-interface often exist behind a functionally intact blood-brain barrier (BBB), and so are shielded from exposure to therapeutic drug concentrations. An ideal glioblastoma treatment needs to engage targets that drive proliferation as well as invasion, with brain penetrant therapies. One such target is the mitotic kinesin KIF11, which can be inhibited with ispinesib, a potent molecularly-targeted drug. Although, achieving durable brain exposures of ispinesib is critical for adequate tumor cell engagement during mitosis, when tumor cells are vulnerable, for efficacy. Our results demonstrate that the delivery of ispinesib is restricted by P-gp and Bcrp efflux at BBB. Thereby, ispinesib distribution is heterogeneous with concentrations substantially lower in invasive tumor rim (intact BBB) compared to glioblastoma core (disrupted BBB). We further find that elacridar—a P-gp and Bcrp inhibitor—improves brain accumulation of ispinesib, resulting in remarkably reduced tumor growth and extended survival in a rodent model of glioblastoma. Such observations show the benefits and feasibility of pairing a potentially ideal treatment with a compound that improves its brain accumulation, and supports use of this strategy in clinical exploration of cell cycle-targeting therapies in brain cancers.

## Introduction

Glioblastoma (GBM), the most common and lethal of primary brain tumors, has two defining phenotypes: uncontrollable proliferation and diffuse infiltration within the brain^1–6^. These features together allow tumor cells to invade regions of brain with an intact blood-brain barrier (BBB), enabling them to proliferate in an environment protected from potentially effective therapeutics. Thus, an ideal therapeutic needs to not only target both proliferation and invasion, but also penetrate the BBB and be retained within tumor-infiltrated brain long enough to kill tumor cells when they are vulnerable to the drug.

The microtubule (MT)-based cytoskeleton is essential for both mitotic spindle function, which drives GBM proliferation, and cell motility, which drives GBM infiltration^7^. Several MT-targeting agents inhibit spindle function, including the taxanes and vinca alkaloids, and these drugs have been successfully used for treating a variety of malignancies^8^. However, MTs are essential for peripheral and central nervous system (PNS, CNS) function, and dose-limiting neurotoxicity has been observed with these MT-targeting drugs^8,9^. This has served as an impetus for developing drugs that target other components of mitotic spindle. One such group are MT-associated molecular motors, referred to as mitotic kinesins, which play multiple roles in mitotic spindle function and cell motility^10,11^. A member of the mitotic kinesins is KIF11 (kinesin family member 11)^12,13^. Over the last twenty years, more than 50 highly specific and potent small-molecule inhibitors of KIF11 have been developed^10^. While these drugs are not neurotoxic^14–17^, they have been disappointing as anti-cancer therapeutics. This has been attributed to the fact that most of the KIF11 inhibitor clinical studies have tested them in solid malignancies which typically have a low proliferative index, using drugs with a relatively short half-life^18^. Mitotic spindle inhibitors are cytotoxic only for cells in the M phase, which constitutes less than 5% of cell cycle duration. Thus, it seems highly unlikely that any appreciable fraction of cells from a tumor with a low proliferative index will be affected by a drug that is only active in M phase, particularly if the drug has a short half-life and can only be dosed intermittently.

KIF11 inhibitors have not been clinically tested in GBM. In spite of the prior disappointing clinical experience, we have shown that KIF11 inhibitors are promising therapeutics in GBM for several reasons^7^. KIF11 is needed for survival of GBM tumor initiating cells (TICs)—the most invasive and chemoresistant cells in this tumor—and is essential for driving both GBM invasion and proliferation^7^. Furthermore, since GBM has a high proliferative index and rarely metastasizes outside the CNS, and given that the brain is essentially a post-mitotic structure, accumulating high doses of a KIF11 inhibitor in brain would essentially target all GBM cells with relatively little CNS toxicity. However, an effective GBM therapeutic must be able to cross the BBB and accumulate in brain at concentrations sufficient to kill tumor cells when they are vulnerable^19,20^. Many small-molecule anti-cancer therapies are prone to active efflux at the BBB by P-glycoprotein (P-gp; also known as ABCB1) and breast cancer resistance protein (Bcrp; also known as ABCG2), and this can severely limit drug distribution to the brain^19,21^. Although the BBB may be relatively compromised in the core of a GBM, infiltrative tumor cells reside behind a functionally intact BBB and result in the establishment of pharmacologic “sanctuaries” that provide sites for tumor recurrence that is inevitable in this disease^22–24^.

Ispinesib (SB-715992) (Supplementary Fig. S1) is a potent and highly specific KIF11 inhibitor, and we have shown that it is active against GBM cells, including treatment resistant TICs, *in vitro* and against orthotopic GBM models *in vivo*^7^. However, even if ispinesib can freely diffuse into the brain, its efficacy could be severely limited if a substantial fraction of the drug is actively pumped out by efflux transporters at the BBB. In order to optimize ispinesib as a GBM therapeutic, we have undertaken a systematic investigation of its pharmacokinetics and pharmacodynamics in orthotopic rodent models of GBM. We find that while ispinesib is a P-gp and Bcrp substrate, its retention and efficacy in GBM can be substantially improved by inhibiting these efflux transporters. Our results support the use of targeting efflux mechanisms as an adjunct to therapeutic development of GBM therapies.

## Results

### Ispinesib is a substrate for the P-gp and Bcrp efflux transporters

**Figure 1:**
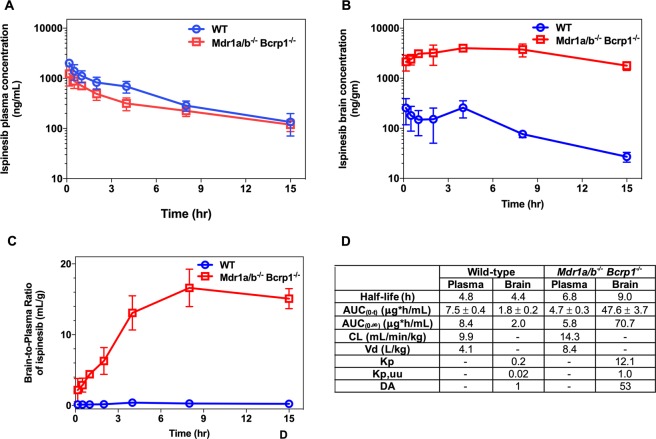
Brain accumulation of ispinesib is limited by active efflux at the BBB. The pharmacokinetic profiles of ispinesib in wild-type and *Mdr1a/b*^*−/−*^ *Bcrp1*^*−/−*^ mice following intravenous bolus dose of 5 mg/kg are shown: (**A**) Plasma concentrations, (**B**) brain concentrations, and (**C**) brain-to-plasma concentration ratios. The pharmacokinetic parameters estimated using non-compartmental analysis (NCA) are listed in the table (**D**). Data represent mean ± S.D., n = 4. The AUCs in the table represent mean ± S.E.M. Abbreviations: AUC_(0-t)_, area under the curve from zero to the time of last measured concentration; CL, clearance; V_d_, volume of distribution; K_p_, the ratio of AUC_(0-t,brain)_ to AUC_(0-t,plasma)_ using total drug concentrations; K_p,uu_, the ratio of AUC_(0-t,brain)_ to AUC_(0-t,plasma)_ using unbound drug concentrations; DA (Distribution Advantage), the ratio of K_p,knockout_ to K_p,wild-type_.

Ispinesib was administered intravenously to Friend leukemia virus strain B (FVB) wild-type mice and to FVB mice deleted for the P-gp and Bcrp efflux transporters (referred to as *Mdr1a/b*^*−/−*^ *Bcrp1*^*−/−*^) in order to determine how these efflux transporters affect brain distribution. The plasma and brain concentration *versus* time profiles and brain-to-plasma ratios following a single intravenous (iv) bolus dose of 5 mg/kg ispinesib are depicted in Fig. 1A–C. At each time point, the brain concentrations are significantly lower than the corresponding plasma concentrations in wild-type mice, while in *Mdr1a/b*^*−/−*^ *Bcrp1*^*−/−*^ mice, they are significantly higher. A summary of the pharmacokinetic parameters is presented in Fig. 1D. The brain-to-plasma AUC ratios (K_p_, *Equation 3*, Supplementary methods) for ispinesib in wild-type and *Mdr1a/b*^*−/−*^ *Bcrp1*^*−/−*^ mice are 0.23 and 12.12, respectively. We further measured free and bound drug in plasma and in brain using rapid equilibrium dialysis (RED) technique. These experiments reveal that ispinesib exhibits a high degree of binding to proteins and cellular constituents. The percentages of unbound drug (*Equations 1 and 2*, Supplementary methods) in plasma and brain are 0.6 ± 0.1% and 0.05 ± 0.02%, respectively. We used the unbound fractions to calculate the unbound concentrations and unbound partition coefficients (K_p,uu_) of ispinesib (*Equation 6*, Supplementary methods). The K_p,uu_ are 0.02 and 1.01 in wild-type and *Mdr1a/b*^*−/−*^ *Bcrp1*^*−/−*^ mice, respectively.

**Figure 2:**
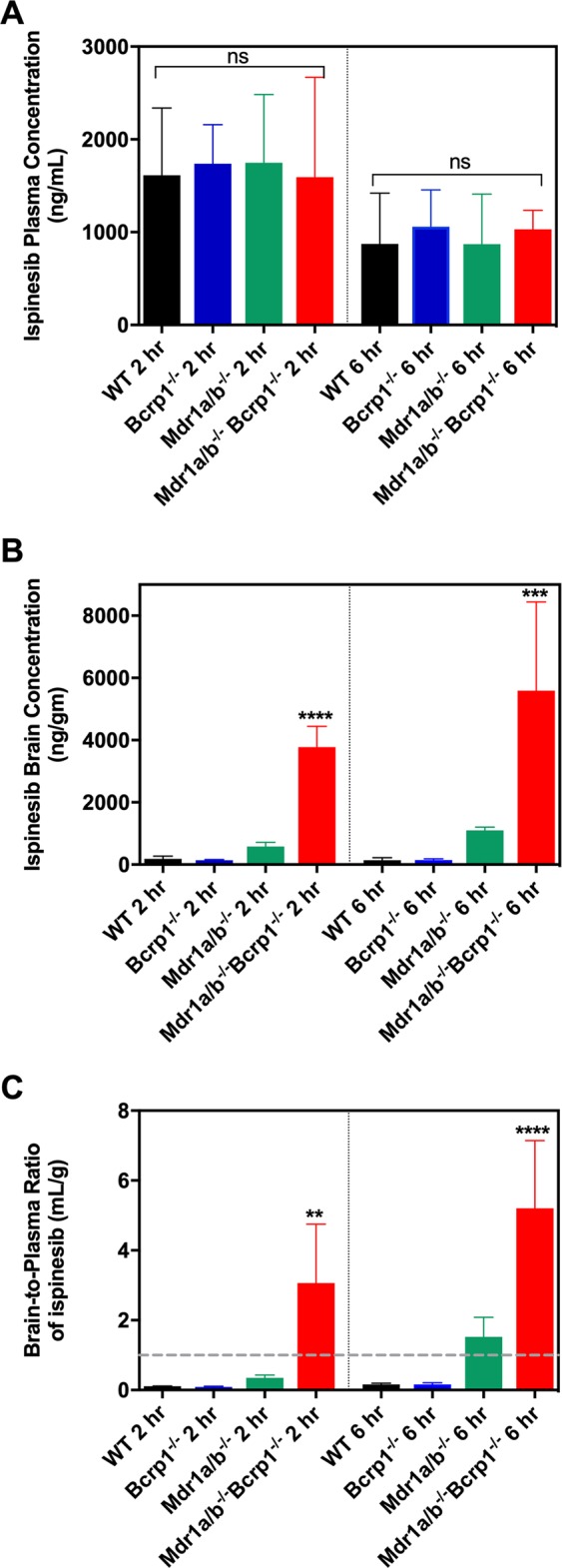
P-gp and Bcrp together restrict the brain distribution of ispinesib. The plasma concentrations (**A**), brain concentrations (**B**), and brain-to-plasma concentration ratios (**C**) at 2 and 6 hours following administration of a single intraperitoneal dose of 10 mg/kg ispinesib to FVB wild-type, *Bcrp1*^*−/−*^, *Mdr1a/b*^*−/−*^ and *Mdr1a/b*^*−/−*^ *Bcrp1*^*−/−*^ mice are depicted. **p < 0.01, ***p < 0.001 and ****p < 0.0001 when compared to the wild-type (WT) groups, for statistical testing by one-way ANOVA. Data represent mean ± S.D., n = 4.

These results demonstrate that ispinesib crosses the BBB but is a substrate for one or both of the P-gp and Bcrp efflux transporters. In order to determine which of these drives ispinesib efflux, we measured ispinesib plasma and brain concentrations, and brain-to-plasma concentration ratios in FVB mice with the following genotypes: wild type, *Mdr1a/b*^*−/−*^ (deleted for only P-gp*), Bcrp1*^*−/−*^ (deleted for only Bcrp), and *Mdr1a/b*^*−/−*^ *Bcrp1*^*−/−*^ (deleted for both) at 2 and 6 hours following intraperitoneal (ip) administration of 10 mg/kg ispinesib. The results are depicted in Fig. 2 and Supplementary Table S1. The plasma concentrations (Fig. 2A) are similar in the four genotypes of mice. However, brain concentrations (Fig. 2B) are significantly higher in *Mdr1a/b*^*−/−*^ *Bcrp1*^*−/−*^ mice compared to wild-type mice. The brain-to-plasma concentration ratios (Fig. 2C) 2 hours after drug administration for wild-type, *Bcrp1*^*−/−*^, *Mdr1a/b*^*−/−*^ and *Mdr1a/b*^*−/−*^ *Bcrp1*^*−/−*^ mice are 0.11, 0.08, 0.35 and 3.07, respectively, while at 6 hours, they are 0.16, 0.15, 1.52 and 5.20, respectively. These results indicate that P-gp and Bcrp play a cooperative role in restricting the brain uptake of ispinesib. We conclude that effective blocking of active efflux of ispinesib at the BBB requires targeting both of these transport proteins.

### Elacridar significantly enhances ispinesib concentrations in brain and in orthotopic GBM

**Figure 3:**
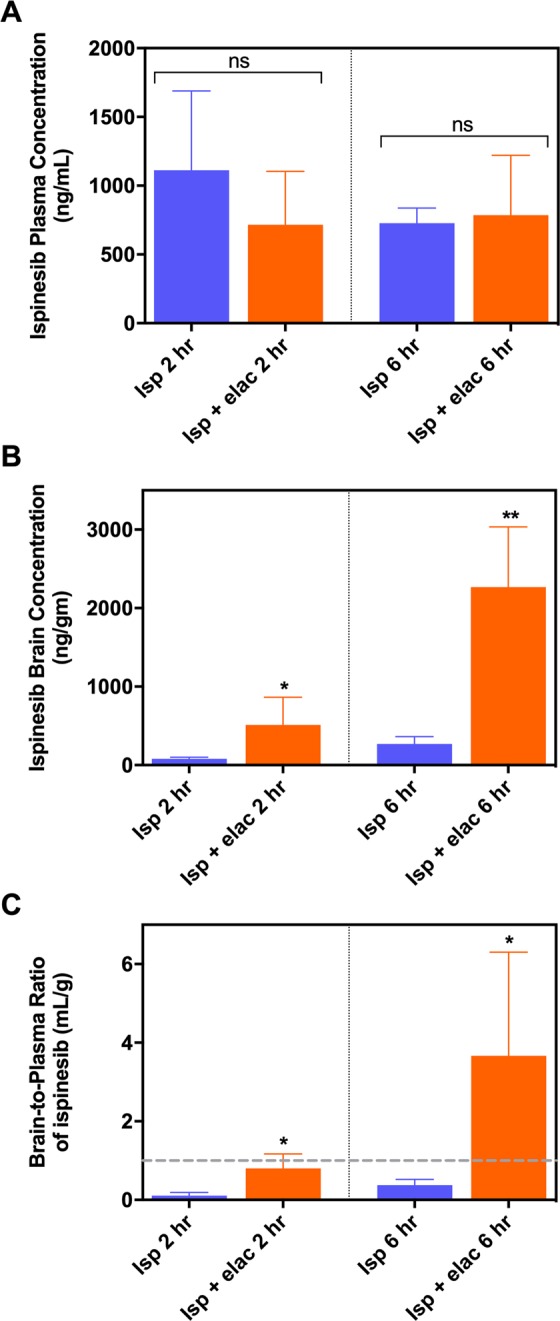
Inhibition of P-gp and Bcrp by elacridar co-administration improves the brain distribution of ispinesib. The plasma concentrations (**A**), brain concentrations (**B**), and brain-to-plasma concentration ratios (**C**) of ispinesib at 2 and 6 hours post dose following a single intraperitoneal administration of 10 mg/kg ispinesib in FVB wild-type mice with or without 10 mg/kg elacridar co-dosing are illustrated. *p < 0.05 and **p < 0.01 for statistical comparison by unpaired t-test. Data represent mean ± S.D., n = 4.

We injected FVB wild-type mice with a single ip dose of 10 mg/kg ispinesib or 10 mg/kg ispinesib simultaneously with 10 mg/kg elacridar, a highly potent and specific inhibitor of P-gp (EC_50_ of 20–200 nM^25,26^) and Bcrp (EC_50_ of about 300 nM^27^), and measured ispinesib concentrations in brain and plasma 2 and 6 hours later. The results are summarized in Fig. 3 and Supplementary Table S2. While the concentration of ispinesib in plasma (Fig. 3A) is unaffected by elacridar at both the time points, the brain concentrations (Fig. 3B) are higher and the brain-to-plasma concentration ratios (Fig. 3C**)** are approximately 10-fold higher with elacridar co-administration.

**Figure 4:**
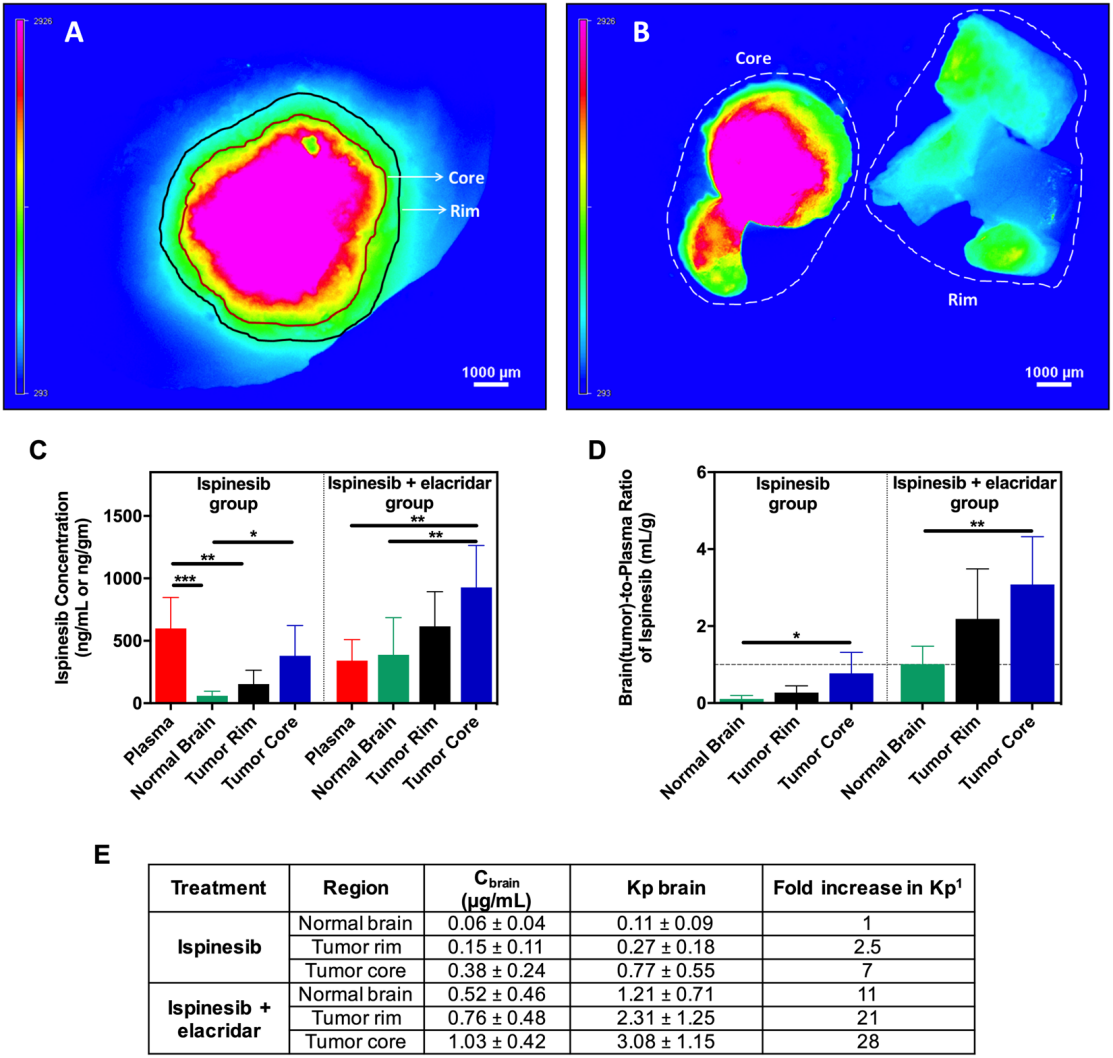
Uptake of ispinesib to regions of intracranial GBM is heterogeneous and is significantly enhanced upon elacridar co-administration. Rats bearing GBM tumors were randomized to receive a single intraperitoneal dose of 10 mg/kg ispinesib with or without simultaneous co-dosing of 10 mg/kg elacridar (n = 9). The blood and brain (tumor-bearing) samples were collected 2 hours post dose, and a fluorescent-guided punching method was employed for isolation of brain regions of interest. The representative images of a brain slice marked with tumor core and tumor rim regions (**A**), and the dissected tissues (**B**) are depicted. The bar graphs represent the concentrations (**C**) and brain(tumor)-to-plasma concentration ratios (**D**) in plasma, regions of tumor and normal brain. The table presents the concentrations, brain(tumor)-to-plasma concentration ratios (K_p_), and fold increase in K_p_ in different regions (**E**). Ispinesib concentrations are significantly higher in ispinesib and elacridar co-dosed group compared to ispinesib group for normal brain (p < 0.05), tumor rim (p < 0.01), and tumor core (p < 0.01). The brain(tumor)-to-plasma ratios of ispinesib are significantly higher in ispinesib and elacridar co-dosed group compared to ispinesib group for normal brain (p < 0.001), tumor rim (p < 0.01), and tumor core (p < 0.01). *p < 0.05, **p < 0.01 and ***p < 0.001 for statistical comparison by one-way ANOVA. Data represent mean ± S.D. Abbreviations: K_p brain_, the ratio of C_brain_ to C_plasma_^1^, fold increase in K_p_ over the K_p_ in normal brain of ispinesib treated group.

We wished to determine how systemically administered ispinesib is distributed within the brain of an orthotopic GBM rodent model, and to evaluate if elacridar co-administration alters this distribution. We generated fluorescent GBMs in 3-day old rat pups by intracerebral injection of a retrovirus encoding for PDGF and TdTomato^28,29^. After an additional 21 days, rat pups were injected ip with a single dose of microemulsion vehicle, 10 mg/kg elacridar, 10 mg/kg ispinesib, or a combination of 10 mg/kg ispinesib and 10 mg/kg elacridar, and they were sacrificed 2 hours later. We employed a fluorescence-guided punching technique (Supplementary Fig. S2) to isolate tumor core, tumor rim (tumor-infiltrated brain), and normal (tumor-free) brain (Fig. 4A,B and Supplementary Fig. S3), measured the concentrations of ispinesib in these samples and in plasma (Fig. 4C), and calculated the corresponding tissue-to-plasma concentration ratios (Fig. 4D). The results show that there is a heterogeneous distribution of ispinesib, with higher drug concentrations in the tumor core compared to the surrounding brain. Furthermore, the uptake/retention of ispinesib is relatively restricted from the invasive tumor rim compared to the tumor core. This trend is similar in both the ispinesib and ispinesib+elacridar treatment groups. However, there is an approximately 10-fold increase in the K_p_ in the tumor rim and normal brain regions and a 4-fold increase in K_p_ in the tumor core when elacridar is co-administered with ispinesib (Fig. 4E). These observations indicate that elacridar co-administration improves the distribution of ispinesib in this orthotopic GBM model, particularly to the invasive edge of the tumor.

### Ispinesib is active against both human and murine GBM cell lines

**Figure 5:**
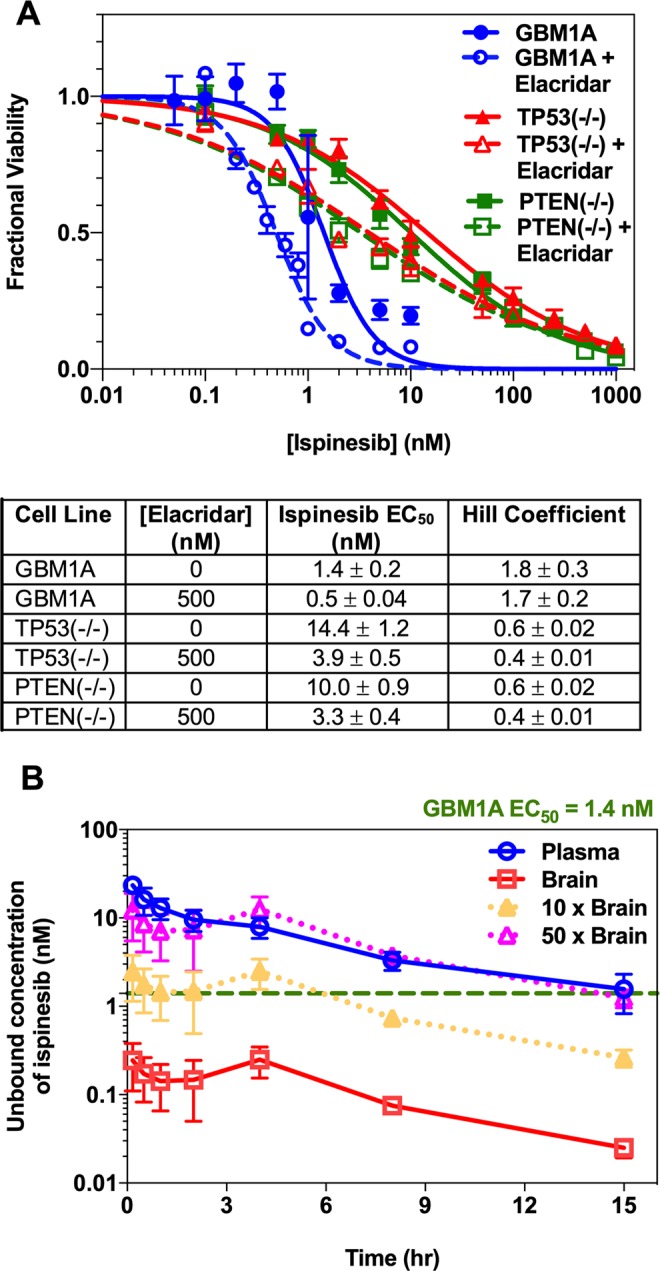
Human and murine glioma cells are sensitive to ispinesib, and unbound concentrations in brain higher than *in vitro* EC_50_ can be achieved by evading efflux transport. (**A**) The responses of human GBM1A, murine TP53(−/−), and murine PTEN(−/−) glioma cell lines to ispinesib alone *(solid lines with closed symbols)* or the combination of ispinesib and elacridar (*dashed lines with open symbols)* are depicted. Data represent mean ± S.D., n = 3 biological replicates. The dose-response data were fit to the Hill equation, and the determined EC_50_s and Hill coefficients are listed in the table. (**B**) The predicted unbound concentration-time profiles of ispinesib in plasma (*solid blue curve*) and brain of FVB wild-type mice following 5 mg/kg intravenous drug administration are depicted. The unbound concentration profiles in brain of wild-type mice (*solid red curve*), with a 10-fold increase in brain concentrations as with elacridar co-administration (*dotted orange curve*), and with a 50-fold increase in brain concentrations as in P-gp and Bcrp deficient mice (*dotted pink curve*) are shown. The unbound concentrations were determined using the *in vivo* concentrations of ispinesib (shown in Fig. 1) and the estimates of unbound fraction (fu) from *in vitro* rapid equilibrium dialysis experiments. The *dashed green line* indicates the experimentally determined *in vitro* EC_50_ in human GBM1A cell line. Data represent mean ± S.D., n = 4.

We measured the efficacy of ispinesib *in vitro* against three GBM cell lines: GBM1A, TP53(−/−), and PTEN(−/−). GBM1A is a human GBM line that has glioma TIC features^30^. TP53(−/−), and PTEN(−/−) are PDGF-driven murine GBM cell lines^31^. Treatment with ispinesib resulted in potent tumor cell killing in all the tested GBM cell lines. The EC_50_s from ispinesib dose-response curves in GBM1A (Fig. 5A, *solid blue line with closed circles*), TP53(−/−) (Fig. 5A, *solid red line with closed triangles*), and PTEN(−/−) (Fig. 5A, *green line with closed squares*) are 1.4 ± 0.2, 14.4 ± 1.2 and 10 ± 0.9 nM, respectively. In addition to being active components of the BBB, both P-gp and Bcrp efflux transporters are frequently expressed in TICs, such as GBM1A^32–34^. We wondered if these two proteins might play a substantial role in modulating intrinsic responsiveness to ispinesib, so we measured ispinesib dose-response relationship of GBM1A cells in the presence of 500 nM elacridar. As Fig. 5A shows, elacridar has at most a modest effect, shifting the EC_50_ of ispinesib for GBM1A (*dashed blue line with open circles*), TP53(−/−) (*dashed red line with open triangles*), and PTEN(−/−) (*dashed green line with open squares*) cells to 0.5 ± 0.04, 3.9 ± 0.5, and 3.3 ± 0.4 nM, respectively.

Subsequently, we wanted to test the efficacy of combining ispinesib with elacridar in an *in vivo* rodent model of GBM. Before performing these studies, however, we compared the predicted unbound concentration-time profiles of ispinesib following 5 mg/kg intravenous administration in FVB wild-type mice with the *in vitro* EC_50_ of ispinesib against GBM1A (Fig. 5B). The comparisons show that the unbound concentrations in the brain (*solid red curve*) are appreciably below the EC_50_ (*dashed green line*) for this drug. Increasing the brain concentrations of ispinesib by 10-fold (*dotted orange curve*), such as would occur with elacridar co-administration, would result in unbound concentrations higher than EC_50_, and thereby allow the brain to be exposed to a therapeutic concentration of the drug for a substantial fraction of time. Increasing the unbound concentration by 50-fold in brain (*dotted pink curve*), such as would occur in P-gp and Bcrp deficient mice, would result in unbound brain concentrations matching the plasma concentrations (*solid blue curve*) and would enhance the time of therapeutic drug exposure substantially further.

### Elacridar significantly improves the efficacy of ispinesib in blocking mitosis in GBM

**Figure 6:**
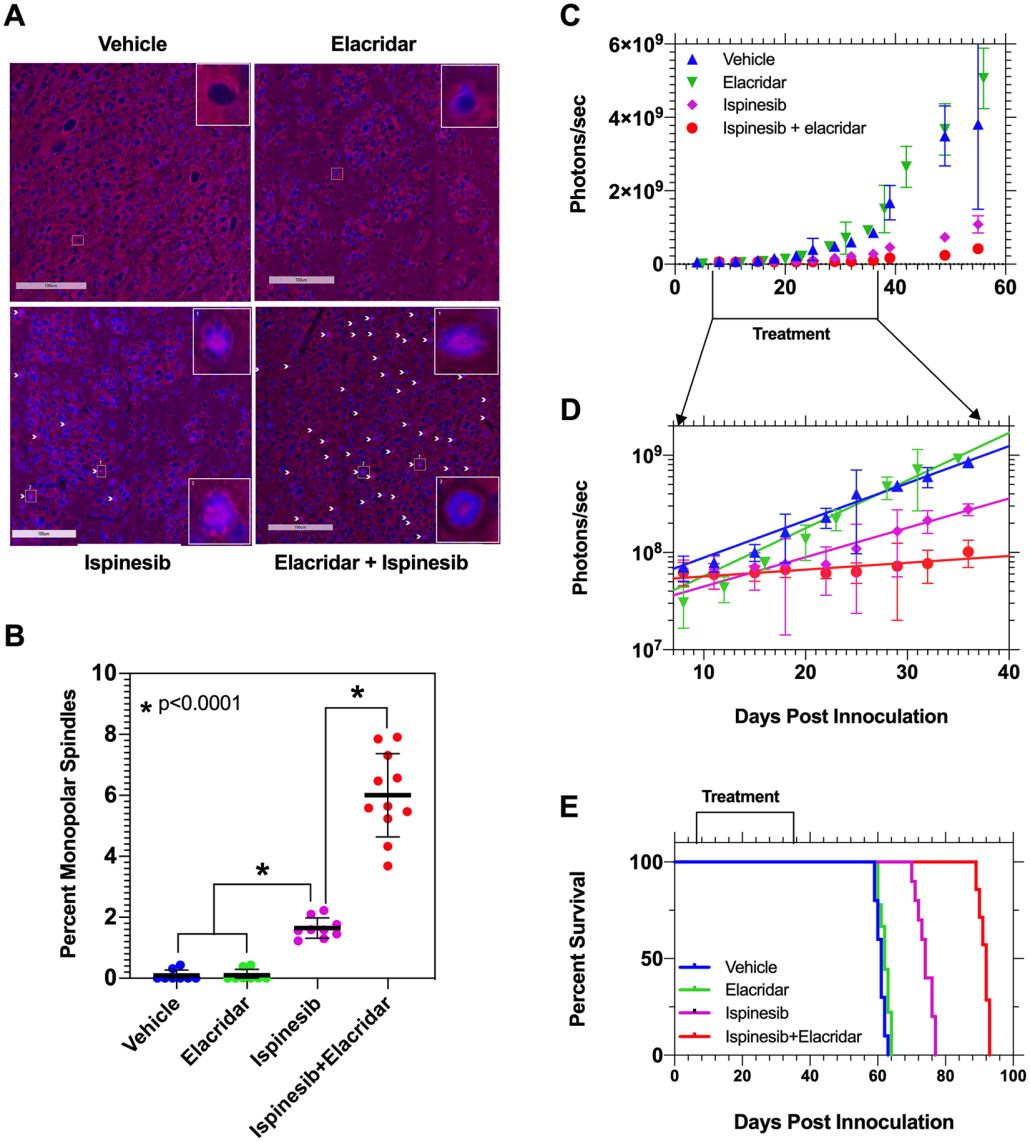
Elacridar co-administration significantly enhances anti-mitotic activity and efficacy of ispinesib in an orthotopic mouse model of GBM. (**A**) Mice with orthotopic GBM were injected with a single intraperitoneal dose of vehicle, 10 mg/kg elacridar, 10 mg/kg ispinesib, or a combination of 10 mg/kg ispinesib and 10 mg/kg elacridar (n = 8–10). At 8 hours, whole brains were collected, stained for tubulin and counter stained for DAPI. The insets in vehicle and elacridar treated groups show bipolar spindle formed during normal mitotic division, DNA (*blue*) in the center surrounded by tubulin (*red*) in periphery. The insets in ispinesib and combination (ispinesib+elacridar treated) groups show collapsed monopolar spindle in a fraction of cells, with tubulin in the center surrounded by DNA-containing chromosomes in periphery. The monopoles are marked with *white arrows*. (**B**) DAPI stained cells and monopolar spindle positive cells were counted, and percent monopolar spindles are depicted. Statistical testing using two tailed t-test. Data represent mean ± S.D., n = 3 biological replicates. (**C**) NSG mice were intracranially injected with luciferase expressing human GBM1A cells. After 7 days, animals were randomized to receive intraperitoneal doses of vehicle, 10 mg/kg elacridar, 10 mg/kg ispinesib, or 10 mg/kg ispinesib and 10 mg/kg elacridar (n = 10) once every 4 days for 28 days (*Treatment*). Tumor growth was monitored by measuring bioluminescence signal, and photon flux was plotted against time. (**D**) The data in (**C**) during treatment period is depicted in an expanded scale. Photon flux for this time period could be fit to a set of single exponential growth equations, revealing doubling times of 7.9 (vehicle), 6.2 (elacridar), 9.4 (ispinesib) and 35.9 (ispinesib+elacridar) days. (E) Kaplan-Meier survival curves that define median survival of 61, 62, 74, and 92 days for vehicle, elacridar, ispinesib, and elacridar+ispinesib treatment, respectively. While differences between vehicle and elacridar do not reach statistical significance (p = 0.07, log rank test), those between vehicle/elacridar and ispinesib, as well as between vehicle/elacridar and ispinesib+elacridar are significant (p < 0.001, log rank test). Likewise, difference in survival between ispinesib and ispinesib+elacridar also reaches statistical significance (p < 0.0001, log rank test).

KIF11 inhibition induces bipolar mitotic spindle to collapse into a monopolar structure, and this histologic hallmark can be used as a surrogate marker for the anti-mitotic effect of ispinesib^7^. We therefore examined if we could enhance this effect by systemically co-administering ispinesib and elacridar to rodents with intracranial GBM. We accomplished this by intraperitoneally injecting a single dose of vehicle, 10 mg/kg elacridar, 10 mg/kg ispinesib, or a combination of 10 mg/kg ispinesib and 10 mg/kg elacridar into NSG mice with orthotopic GBM1A tumors, sacrificing animals after 8 hours, excising brains, and staining sections through the core of the tumor for tubulin and DNA. We counted the number of cells with monopolar spindles in each of these four conditions to determine the fraction of cells with monopoles (Fig. 6A). The insets in the vehicle and elacridar treated brains show the normal distribution of DNA (*blue*) in the center, surrounded by tubulin (*red*) in the periphery. By contrast, ispinesib administration produces occasional monopoles, with DNA-containing chromosomes in the periphery surrounding a center of tubulin (*white arrows*). Co-administration of elacridar with ispinesib markedly increased the fraction of monopoles. This is quantified in Fig. 6B, which demonstrates that elacridar significantly enhances the ability of ispinesib to produce the desired pharmacodynamic effect.

### Elacridar significantly enhances the efficacy of systemically administered ispinesib in a rodent model of GBM

We injected 500,000 luciferase expressing GBM1A cells into the white matter of NSG recipient mice and initiated treatment 7 days later with vehicle, 10 mg/kg ip elacridar, 10 mg/kg ip ispinesib, or a combination of 10 mg/kg ispinesib and 10 mg/kg elacridar ip, once every 4 days for a total of 28 days. We monitored the increase in bioluminescence (BLI) signal to assess the kinetics of tumor growth and followed animals for survival, and results are depicted in Fig. 6C–E. Figure 6C depicts photon flux *versus* time during the course of the experiment, with the period of drug treatment (*days 7–37 post tumor inoculation*) indicated. Figure 6D illustrates a plot of BLI over days 7–37, with data fit to single exponential growth equations. This reveals tumor doubling times of 7.9 (vehicle), 6.2 (elacridar), 9.4 (ispinesib) and 35.9 (ispinesib + elacridar) days. The mice in this experiment were also followed for survival after discontinuing treatment on day 37, and Kaplan Meier survival curves are illustrated in Fig. 6E. The median survival for vehicle, elacridar, ispinesib, and elacridar+ispinesib treated groups are 61, 62, 74, and 92 days, respectively. While treatment with ispinesib alone prolongs survival compared to vehicle or elacridar (p < 0.0001, log rank test), combined therapy with elacridar and ispinesib is clearly superior to ispinesib alone (p < 0.0001, log rank test). These results demonstrate a significant improvement in efficacy when elacridar was co-dosed to enhance the brain accumulation of ispinesib. Also, the combination of ispinesib and elacridar in the above dosing regimen was well tolerated without any toxicity related death events.

## Discussion

Tumor heterogeneity has been a defining feature for GBM since the early histopathology studies of the 19^th^ century^35^. More recently, this heterogeneity has been documented to occur at the genetic, epigenetic, and gene expression levels, both from one GBM tumor to another, as well as within a given GBM^36–38^. However, heterogeneity also occurs at the level of cell biology, as GBM cells have also been observed to demonstrate two distinct phenotypes—one characterized by high proliferation and the other by high dispersion. Furthermore, we and others have shown that these two phenotypes are reciprocally related— inhibiting one activates the other^7,39^. Thus, an ideal GBM therapeutic needs to inhibit a target that drives both phenotypes simultaneously and be active against TICs and nonTICs alike. Our prior study^7^ has shown that ispinesib addresses these requirements. However, to be clinically effective, ispinesib must also cross the BBB and be retained at sustained therapeutic concentrations within both tumor and surrounding brain. Our previous studies with other anti-cancer agents that are active against GBM *in vitro* and in *in vivo* flank models have shown that restricted brain delivery can render these promising compounds ineffective in orthotopic GBM^40–42^. A key contributor to this problem is P-gp and Bcrp-mediated active efflux at the BBB. In this study, we have examined whether we can realize the potential of a promising GBM therapeutic, ispinesib, by improving its delivery to and retention within both brain and tumor.

The tightly regulated structure of the BBB is known to limit the delivery of a wide range of CNS therapeutics to the brain. For this reason, we sought to examine the brain distribution of ispinesib in FVB mice. The results shown in Fig. 1 suggest severely restricted delivery of ispinesib to the brain, as reflected from the brain-to-plasma partition coefficient (K_p_) of 0.23 and unbound partition coefficient (K_p,uu_) of 0.02 in wild-type mice. A major factor responsible for restricting the brain delivery of therapies is the gatekeeper function of the P-gp and Bcrp efflux transporters at the BBB^19,20^. We therefore performed additional studies in transporter-deleted mice to examine the influence of P-gp and Bcrp on the brain delivery of ispinesib. The K_p_ and K_p,uu_ in P-gp and Bcrp deficient mice were 12.12 and 1.01, respectively, which are substantially higher than the corresponding values in wild-type mice (Fig. 1). These results demonstrate that P-gp and Bcrp-mediated drug efflux plays a significant role in limiting the brain accumulation of ispinesib (Fig. 2). Together, the brain distribution studies in FVB mice show that ispinesib has poor brain retention due to active efflux by P-gp and Bcrp transport systems at the BBB.

Our results with elacridar, a potent non-toxic inhibitor of P-gp and Bcrp, highlight the importance of inhibiting efflux transport in order to improve brain delivery of promising therapeutics. Our results demonstrate a 10-fold enhancement in the brain delivery of ispinesb (Fig. 3) with co-administration of elacridar implying that targeting drug efflux transport at the BBB enhances the brain accumulation of ispinesib. There have been reports of spatial heterogeneity in drug uptake to brain tumors, with severely limited drug accumulation in certain tumor regions residing behind an intact BBB^23,24,43^. Such restricted delivery can result in the establishment of a protected pharmacological sanctuary for the tumors that can therefore grow unimpeded within the CNS. We therefore measured the regional drug distribution in a preclinical model of GBM, and also evaluated if ispinesib accumulation in the regions of tumor with an intact BBB can be improved with elacridar co-administration. The results depicted in Fig. 4 reveal regional variability in ispinesib delivery with higher accumulation in the tumor core, where the BBB is relatively compromised, and with lower accumulation in the invasive tumor rim, where the BBB is more often intact. This confirms that systemic delivery of ispinesib to treat GBM would likely lead to early disease relapse, due to inadequate drug concentrations at the tumor/brain interface. We therefore tested the impact of elacridar co-administration on the delivery of ispinesib to regions of GBM. Our observations, depicted in Fig. 4, show that there is an improvement in the delivery of ispinesib to the tumor, not only in regions where the BBB is already defective, but in the invasive margin of the tumor as well.

Our brain distribution studies prompted us to explore if we could translate this enhanced delivery to improved efficacy in mice with orthotopic GBMs. We began by comparing the *in vitro* cytotoxic concentrations of ispinesib in a GBM cell line (Fig. 5A) with the unbound brain concentration-time profile in wild-type mice. This reveals that the free concentration of ispinesib in the brain following an intravenous dose of 10 mg/kg is lower than the *in vitro* EC_50_ in GBM1A (*solid red curve*, Fig. 5B). However, our results predict that co-administration of elacridar should appreciably enhance the fraction of time that brain and GBM are exposed to therapeutic drug concentrations (*dotted orange curve*, Fig. 5B), which in turn would enhance the probability that sufficient drug would be present to kill cells when they are vulnerable, in M phase. This prompted us to examine the effect of elacridar co-administration on monopolar spindle formation (Fig. 6A,B), *in vivo* tumor growth kinetics, and survival (Fig. 6C–E) in an orthotopic GBM1A mouse model. We find that elacridar co-administration markedly increases the frequency of monopolar spindles—a surrogate marker for anti-mitotic activity of ispinesib *in situ*. Our *in vivo* efficacy studies build on this finding by demonstrating that while ispinesib by itself slows tumor growth and improves survival in an *in vivo* GBM model, these effects can be markedly enhanced by co-administration of elacridar. These outcomes show that improving the brain accumulation of ispinesib results in superior *in vivo* target engagement and efficacy in an orthotopic model of GBM.

While the BBB passively prevents the entry of hydrophilic drugs into the CNS, it is also capable of actively extruding hydrophobic small molecules, primarily through the action of two ABC transporters, P-gp and Bcrp^19,20^. Together, these features of the BBB present a major obstacle to the development of effective therapies for GBM. Although the BBB is heterogeneously compromised within a GBM, viable tumor cells are found in the brain-infiltrative margin where the BBB is intact, and this feature provides a therapeutic sanctuary that drives the inevitable recurrence of disease^24^. This highlights the general principle that efforts to identify new GBM targets and develop new drugs against such targets are in vain if they cannot be translated into therapies that reach and remain at the site of tumor growth and invasion. While the ability of the BBB to shield GBM from potentially effective therapeutics applies to any drug, it is particularly problematic for therapeutics that are only active during distinct phases of the cell cycle. Inhibitors of the mitotic kinesin KIF11 illustrate this point. The motivation for developing these inhibitors was the presumption that they would be devoid of the neurotoxicity seen with microtubule-directed anti-mitotics, and the clinical experience with these drugs has confirmed this^14–17^. However, KIF11 inhibitors are effective only when they are present in therapeutic concentrations during M phase, which accounts for only about 5% of cell cycle time^10^. Thus, for a KIF11 inhibitor to be therapeutically active in GBM, it needs not only to cross the BBB, but be retained within both brain and tumor at effective concentrations for considerable periods of time in order to target a substantial proportion of tumor cells when they are vulnerable.

Although we had previously shown that the KIF11 inhibitor ispinesib is capable of crossing the BBB and prolonging survival in a patient-derived xenograft model of GBM, we suspected that active extrusion of this hydrophobic therapeutic might limit its ability to control this disease^7^. Our current study confirms this suspicion. Ispinesib is indeed a substrate for P-gp and Bcrp transporters, which together reduce drug exposure *in situ* to sub-therapeutic concentrations. We have gone on to demonstrate that co-administration of elacridar, a potent and specific third generation inhibitor of both efflux transporters^25,27^ can significantly enhance: i) brain concentrations of ispinesib, ii) the frequency of monopolar spindles—a surrogate marker for KIF11 inhibition; and iii) survival over ispinesib alone in an orthotopic xenograft model implanted with human GBM. Our results highlight the importance of blocking drug efflux transporters in GBM when sustained concentrations of drug are needed for a therapeutic effect. Although safety concerns have limited the clinical testing of efflux transport inhibitors^44,45^, we find that the combination of ispinesib and elacridar is well tolerated and does not lead to any toxicity related deaths. In addition, elacridar has been safely administered to patients in recent studies, and the development of an oral formulation of elacridar has revived interest in testing this compound in the clinic^46,47^. In short, our findings indicate that a GBM therapeutic which targets both of the malignant phenotypes of this disease—brain invasion and proliferation—can be made much more effective by simultaneously targeting the BBB efflux transporters in a manner that does not enhance drug toxicity. We believe that our approach in this study is applicable to a wide variety of other GBM therapies, and should be considered in designing both pre-clinical as well as clinical trials of promising drugs for the treatment of GBM.

## Materials and Methods

### Chemicals

Ispinesib, N-(3-aminopropyl)-N-[(1 R)-1-(3-benzyl-7-chloro-4-oxoquinazolin-2-yl)-2-methylpropyl]-4-methylbenzamide, was purchased from Selleck Chemicals (Houston, TX). Elacridar [GF-120918, N-[4-[2-(6,7-dimethoxy-3,4-dihydro-1H-isoquinolin-2-yl)ethyl]phenyl]-5-methoxy-9-oxo-10H-acridine-4-carboxamide] was purchased from Toronto Research Chemicals Inc. (Ontario, Canada).

### *In vitro* assay for determination of unbound (free) fractions

Rapid equilibrium dialysis was used to determine unbound fractions with modifications^48,49^ to manufacturer’s protocol (Thermo Fisher Scientific). Mouse plasma and brain homogenates (in 3 volumes of PBS, w/v) were spiked with 0.1 mg/mL ispinesib to obtain final concentrations of 1 µM. 300 µL of drug-spiked matrices were placed in donor chamber, and 500 µL of PBS was placed in receiver chamber of inserts (8 kDa MWCO), in triplicates. The base plate with inserts was incubated on Bioshaker at 37 °C and 1000 rpm for 4 hours. Ispinesib concentrations in samples were analyzed by liquid-chromatography tandem mass-spectrometry (LC-MS/MS). Additional details are described in the supplemental methods.

### *In vitro* cytotoxicity assay

TP53(−/−) and PTEN(−/−) cells were seeded into 96-well plates (pre-coated with fibronectin) at a density of 5,000 cells per well in DMEM media with 0.5% FBS, 1% nitrogen supplement, 10 ng/ml of FGF and 10 ng/ml of PDGFAA. Human GBM1A cells were seeded into 96 well plates (pre-coated with laminin) in DMEM media with 1% neuroPlex supplement, 20 ng/ml of EGF and 20 ng/ml of FEF. After 24 hours (~70% confluency), cells were exposed to ispinesib (n = 10 wells per concentration) from 0.025–1000 nM with or without 500 nM elacridar co-treatment. The plates were incubated for 72 hours, cell viability measured using CellTiter-Glo ATP-based assay, and read on a luminometer. The dose-response data were fit to the Hill equation.

### *In vivo* studies

All studies conducted were in compliance with the guidelines for the Care and Use of Laboratory Animals (NIH), and approved by Institutional Animal Care and Use Committee (IACUC) at University of Minnesota or Mayo Clinic Foundation. Animals were housed in standard 12-hour light/dark cycle with unlimited access to food and water. Retrovirus production, intracranial injection methods, and establishment of primary cell lines were performed as described previously^28^.

### Pharmacokinetics following intravenous and intraperitoneal administration of ispinesib

A single intravenous bolus dose of 5 mg/kg ispinesib (vehicle: ethanol, Tween 80 and distilled water in volume ratio of 20:2.5:77.5) was administered to FVB wild-type and *Mdr1a/b*^*−/−*^ *Bcrp1*^*−/−*^ mice. This was followed by collection of whole blood and brain samples at 0.17, 0.5, 1, 2, 4, 8 and 15 hours post-dose, and blood samples were centrifuged to separate plasma for further analysis. In another study, a single intraperitoneal dose of 10 mg/kg ispinesib (microemulsion vehicle: Cremophor EL, Carbitol, Captex 355 and distilled water in volume ratio of 20:10:3:67) was dosed in wild-type, *Mdr1a/b*^*−/−*^*, Bcrp1*^*−/−*^, and *Mdr1a/b*^*−/−*^ *Bcrp1*^*−/−*^ mice. At 2 and 6 hours following administration of ispinesib, whole blood and brain samples were harvested, and blood samples were centrifuged for plasma separation. Additional details on the conduct of *in vivo* pharmacokinetic studies are described in the supplementary methods. The drug concentrations in all samples were measured by a specific and sensitive LC-MS/MS assay for ispinesib.

### Brain distribution of ispinesib with and without co-administration of elacridar

FVB wild-type mice received a single dose of 10 mg/kg ip ispinesib with or without simultaneous co-administration of 10 mg/kg ip elacridar, both in microemulsion vehicle. Ispinesib and elacridar (half-life of 3.2 hrs following ip dosing^50^) have comparable half-lives and so were dosed together. Blood and brain specimens were harvested at 2 and 6 hours post-dose.

### Spatial distribution of ispinesib in a rat model of GBM

The GBM tumors were induced by stereotactic injection of a PDGF-IRES-tdTomato retrovirus in rat pups^28^, using postnatal day 3 (P3) neonatal Sprague-Dawley rats. Rats were randomized (n = 9) to receive single dose of 10 mg/kg ip ispinesib with or without simultaneous co-administration of 10 mg/kg ip elacridar on day 21 following intracranial injections. Blood and brain (tumor-bearing) samples were collected at 2 hours post-dose, and whole brains were immediately flash frozen.

A fluorescence-guided punch biopsy technique (Supplementary Fig. S2) was developed and employed for isolation of tumor core, tumor rim (brain adjacent to tumor, BAT) and normal (non-tumor) brain regions from brain samples. Briefly, an acrylic adult rat brain matrix (WPI) was used to obtain thick coronal brain sections (1–2 mm thick) through the Td-Tomato labelled tumors. The tumor regions were identified by relative fluorescence signal (Nikon AZ100M microscope), and biospy punches with varying diameters were utilized to isolate tumor core (tumor region with fluorescence signal 5-fold or higher relative to background signal) and rim (region adjacent to tumor core with fluorescence signal 3 to 5-fold higher relative to background) (Supplementary Fig. S3). The samples from individual brains were pooled together for analysis of ispinesib concentrations by LC-MS/MS.

### Pharmacodynamics of ispinesib in a mouse model of GBM

Female NSG mice were intracerebrally injected with 500,000 GBM1A cells at coordinates X = 1.5 mm, Y = 1.5 mm and Z = 2.5 mm relative to the bregma, and tumor growth was monitored by bioluminescence imaging. After 4 weeks (tumor size of about 2 × 10^9^ photons/sec), animals were randomized to receive single ip dose of microemulsion vehicle, 10 mg/kg elacridar, 10 mg/kg ispinesib, or a combination of 10 mg/kg ispinesib and 10 mg/kg elacridar (n = 8–10). At 8 hours following treatment, mice were perfused and whole brains were isolated. The samples were processed for immunofluorescence by staining for tubulin with an alpha-tubulin antibody (Cell signaling, Cat#2125) and counter staining with DAPI for visualizing nuclei. Images were scanned and counted for cells with monopolar spindles.

### In vivo efficacy of ispinesib in a mouse model of GBM

Female NSG mice were intracranially implanted with 500,000 luciferase expressing GBM1A cells (transduced with lentiviral particles expressing GFP-Luciferase). After 7 days, animals were randomized into four groups and ip administered with microemulsion vehicle, 10 mg/kg elacridar, 10 mg/kg ispinesib, or a combination of 10 mg/kg ispinesib and 10 mg/kg elacridar, once every 4 days for 28 days (n = 10). The dosing schedule of once every 4 days was based on our previously publication with ispinesib^7^. Tumor growth was monitored by bioluminescence imaging and animals were followed for survival.

### LC-MS/MS analysis

A detailed description is provided in the supplemental methods. In brief, all samples were spiked with dasatinib as internal standard. After extraction in ethyl acetate, organic supernatant was dried under nitrogen, reconstituted in mobile phase (55:45 of 1 mM ammonium formate with 0.1% formic acid: acetonitrile), and injected onto a Phenomenex Synergi 4 µ Polar-RP 80 A column. The m/z transitions were 517.20–246.96 for ispinesib and 488.21–400.99 for dasatinib (positive-ionization mode).

### Statistical and data analysis

The details of pharmacokinetic data analysis, calculations and statistical testing are described in the supplemental methods.

## Supplementary information

Supplementary information.

## References

[1] Chiesa-Vottero AG, Rybicki LA, Prayson RA (2003). Comparison of proliferation indices in glioblastoma multiforme by whole tissue section vs tissue microarray. Am J Clin Pathol.

[2] Stoyanov GS, Dzhenkov DL, Kitanova M, Donev IS, Ghenev P (2017). Correlation Between Ki-67 Index, World Health Organization Grade and Patient Survival in Glial Tumors With Astrocytic Differentiation. Cureus.

[3] Matsukado Y, Maccarty CS, Kernohan JW (1961). The growth of glioblastoma multiforme (astrocytomas, grades 3 and 4) in neurosurgical practice. J Neurosurg.

[4] Demuth T, Berens ME (2004). Molecular mechanisms of glioma cell migration and invasion. J Neurooncol.

[5] Cuddapah VA, Robel S, Watkins S, Sontheimer H (2014). A neurocentric perspective on glioma invasion. Nat Rev Neurosci.

[6] Mastronardi L, Guiducci A, Puzzilli F, Ruggeri A (1999). Relationship between Ki-67 labeling index and survival in high-grade glioma patients treated after surgery with tamoxifen. J Neurosurg Sci.

[7] Venere M (2015). The mitotic kinesin KIF11 is a driver of invasion, proliferation, and self-renewal in glioblastoma. Sci Transl Med.

[8] Wood KW, Cornwell WD, Jackson JR (2001). Past and future of the mitotic spindle as an oncology target. Curr Opin Pharmacol.

[9] Canta A, Chiorazzi A, Cavaletti G (2009). Tubulin: a target for antineoplastic drugs into the cancer cells but also in the peripheral nervous system. Curr Med Chem.

[10] Rath O, Kozielski F (2012). Kinesins and cancer. Nat Rev Cancer.

[11] Cross RA, McAinsh A (2014). Prime movers: the mechanochemistry of mitotic kinesins. Nat Rev Mol Cell Biol.

[12] Sarli V, Giannis A (2008). Targeting the kinesin spindle protein: basic principles and clinical implications. Clin Cancer Res.

[13] Wojcik EJ (2013). Kinesin-5: cross-bridging mechanism to targeted clinical therapy. Gene.

[14] Blagden SP (2008). A phase I trial of ispinesib, a kinesin spindle protein inhibitor, with docetaxel in patients with advanced solid tumours. Br J Cancer.

[15] Souid AK (2010). A pediatric phase I trial and pharmacokinetic study of ispinesib: a Children’s Oncology Group phase I consortium study. Pediatr Blood Cancer.

[16] Burris HA (2011). A phase I study of ispinesib, a kinesin spindle protein inhibitor, administered weekly for three consecutive weeks of a 28-day cycle in patients with solid tumors. Invest New Drugs.

[17] Gomez HL (2012). Phase I dose-escalation and pharmacokinetic study of ispinesib, a kinesin spindle protein inhibitor, administered on days 1 and 15 of a 28-day schedule in patients with no prior treatment for advanced breast cancer. Anticancer Drugs.

[18] Komlodi-Pasztor E, Sackett DL, Fojo AT (2012). Inhibitors targeting mitosis: tales of how great drugs against a promising target were brought down by a flawed rationale. Clin Cancer Res.

[19] Agarwal S, Sane R, Oberoi R, Ohlfest JR, Elmquist WF (2011). Delivery of molecularly targeted therapy to malignant glioma, a disease of the whole brain. Expert Rev Mol Med.

[20] Gampa G, Vaidhyanathan S, Sarkaria JN, Elmquist WF (2017). Drug delivery to melanoma brain metastases: Can current challenges lead to new opportunities?. Pharmacol Res.

[21] Gampa G (2016). Challenges in the delivery of therapies to melanoma brain metastases. Curr Pharmacol Rep.

[22] Jain RK (2007). Angiogenesis in brain tumours. Nat Rev Neurosci.

[23] Osswald M (2016). Impact of Blood-Brain Barrier Integrity on Tumor Growth and Therapy Response in Brain Metastases. Clin Cancer Res.

[24] Sarkaria JN (2018). Is the blood-brain barrier really disrupted in all glioblastomas? A critical assessment of existing clinical data. Neuro Oncol.

[25] Rautio J (2006). *In vitro* p-glycoprotein inhibition assays for assessment of clinical drug interaction potential of new drug candidates: a recommendation for probe substrates. Drug Metab Dispos.

[26] Keogh JP, Kunta JR (2006). Development, validation and utility of an *in vitro* technique for assessment of potential clinical drug-drug interactions involving P-glycoprotein. Eur J Pharm Sci.

[27] Ahmed-Belkacem A (2005). Flavonoid structure-activity studies identify 6-prenylchrysin and tectochrysin as potent and specific inhibitors of breast cancer resistance protein ABCG2. Cancer Res.

[28] Assanah MC (2009). PDGF stimulates the massive expansion of glial progenitors in the neonatal forebrain. Glia.

[29] Beadle C (2008). The role of myosin II in glioma invasion of the brain. Mol Biol Cell.

[30] Tilghman J (2016). Regulation of Glioblastoma Tumor-Propagating Cells by the Integrin Partner Tetraspanin CD151. Neoplasia.

[31] Sonabend AM (2014). The transcriptional regulatory network of proneural glioma determines the genetic alterations selected during tumor progression. Cancer Res.

[32] Matsumoto T, Tani E, Kaba K, Shindo H, Miyaji K (1991). Expression of P-glycoprotein in human glioma cell lines and surgical glioma specimens. J Neurosurg.

[33] Loscher W, Potschka H (2005). Role of drug efflux transporters in the brain for drug disposition and treatment of brain diseases. Prog Neurobiol.

[34] van Tellingen O (2015). Overcoming the blood-brain tumor barrier for effective glioblastoma treatment. Drug Resist Updat.

[35] Inda MM, Bonavia R, Seoane J (2014). Glioblastoma multiforme: a look inside its heterogeneous nature. Cancers (Basel).

[36] Coons SW, Johnson PC, Shapiro JR (1995). Cytogenetic and flow cytometry DNA analysis of regional heterogeneity in a low grade human glioma. Cancer Res.

[37] Cancer Genome Atlas Research, N. (2008). Comprehensive genomic characterization defines human glioblastoma genes and core pathways. Nature.

[38] Patel AP (2014). Single-cell RNA-seq highlights intratumoral heterogeneity in primary glioblastoma. Science.

[39] Berens ME, Giese A (1999). “…those left behind.” Biology and oncology of invasive glioma cells. Neoplasia.

[40] Agarwal S (2012). Active efflux of Dasatinib from the brain limits efficacy against murine glioblastoma: broad implications for the clinical use of molecularly targeted agents. Mol Cancer Ther.

[41] Parrish KE (2015). Efflux transporters at the blood-brain barrier limit delivery and efficacy of cyclin-dependent kinase 4/6 inhibitor palbociclib (PD-0332991) in an orthotopic brain tumor model. J Pharmacol Exp Ther.

[42] Kim M (2018). Efficacy of the MDM2 Inhibitor SAR405838 in Glioblastoma Is Limited by Poor Distribution Across the Blood-Brain Barrier. Mol Cancer Ther.

[43] Lockman PR (2010). Heterogeneous blood-tumor barrier permeability determines drug efficacy in experimental brain metastases of breast cancer. Clin Cancer Res.

[44] Sandler A (2004). A Phase I trial of a potent P-glycoprotein inhibitor, zosuquidar trihydrochloride (LY335979), administered intravenously in combination with doxorubicin in patients with advanced malignancy. Clin Cancer Res.

[45] Kuppens IE (2007). A phase I, randomized, open-label, parallel-cohort, dose-finding study of elacridar (GF120918) and oral topotecan in cancer patients. Clin Cancer Res.

[46] Sawicki E (2017). Clinical pharmacokinetics of an amorphous solid dispersion tablet of elacridar. Drug Deliv Transl Res.

[47] Verheijen RB (2018). Molecular Imaging of ABCB1 and ABCG2 Inhibition at the Human Blood-Brain Barrier Using Elacridar and (11)C-Erlotinib PET. J Nucl Med.

[48] Friden M, Gupta A, Antonsson M, Bredberg U, Hammarlund-Udenaes M (2007). *In vitro* methods for estimating unbound drug concentrations in the brain interstitial and intracellular fluids. Drug Metab Dispos.

[49] Kalvass JC, Maurer TS (2002). Influence of nonspecific brain and plasma binding on CNS exposure: implications for rational drug discovery. Biopharm Drug Dispos.

[50] Sane R, Mittapalli RK, Elmquist WF (2013). Development and evaluation of a novel microemulsion formulation of elacridar to improve its bioavailability. J Pharm Sci.

