# Supplementary material for: Enhancing Brain Retention of a KIF11 Inhibitor Significantly Improves its Efficacy in a Mouse Model of Glioblastoma
Source: Sci Rep. 2020 Apr 16;10:6524. doi: 10.1038/s41598-020-63494-7 (PMC7162859; doi:10.1038/s41598-020-63494-7)
Supplement: Supplementary file 1 — Supplementary information. [file 41598_2020_63494_MOESM1_ESM.docx]

**Enhancing Brain Retention of a KIF11 Inhibitor Significantly Improves its Efficacy in a Mouse Model of Glioblastoma**

Gautham Gampa^1#^, Rajappa S. Kenchappa^2#^, Afroz S. Mohammad^1^, Karen E. Parrish^1^, Minjee Kim^1^, James F. Crish^3^, Amanda Luu^2^, Rita West^2^, Alfredo Quinones Hinojosa^4^, Jann N. Sarkaria^5^, Steven S. Rosenfeld^2^*, and William F. Elmquist^1^*

^1^Brain Barriers Research Center, Department of Pharmaceutics, College of Pharmacy, University of Minnesota, Minneapolis, MN

^2^Department of Cancer Biology, Mayo Clinic, Jacksonville, FL

^3^Department of Cancer Biology, Cleveland Clinic, Cleveland, OH

^4^Department of Neurologic Surgery, Mayo Clinic, Jacksonville, FL

^5^Department of Radiation Oncology, Mayo Clinic, Rochester, MN

^#^These authors contributed equally

*Corresponding authors

**SUPPLEMENTARY MATERIALS**

**SUPPLEMENTARY METHODS**

### *In vitro rapid equilibrium dialysis*

The unbound fractions of ispinesib in plasma and brain were determined by using rapid equilibrium dialysis (RED) technique, with some alterations ^1,2^ to manufacturer’s protocol (Thermo Fisher Scientific). The methodology employed for these experiments was similar to that described previously ^3,4^. RED experiments were initially conducted at 1, 2, 5 and 10 µM concentrations of ispinesib with 4 and 6 hour time points. These preliminary experiments showed that the unbound fractions of ispinesib were linear up to 2 µM with equilibrium established by 4 hours. Consequently, unbound fractions at 1 µM concentration and 4 hr time point were utilized for estimation of Kp,uu and free concentrations of ispinesib. Briefly, the procedure involved the use of a RED base plate and single-use RED inserts with a dialysis membrane having a molecular weight cut off (MWCO) of 8 kDa. Aliquots of fresh plasma and brain homogenates (prepared in 3 volumes of PBS, w/v) from FVB mice were spiked with 0.1 mg/mL ispinesib (in DMSO) to obtain final concentrations of 1 µM. A 300 µL aliquot of 1 µM ispinesib spiked matrix was placed in sample chamber (donor), and 500 µL of phosphate buffered saline (PBS) was placed in buffer chamber (receiver) of RED inserts, in triplicates. The inserts were placed in a RED base plate, assembly was covered with a sealing tape and incubated on a Bioshaker (MBR-022UP, Taitec Corporation) maintained at 37^o^C and 1000 rpm for 4 hours. The samples collected from the donor and receiver chambers after dialysis for 4 hours were stored at -80°C and analyzed for ispinesib concentrations by LC-MS/MS analysis.

### *Animals*

Friend leukemia virus strain B (FVB) wild-type (WT), P-gp knockout (*Mdr1a/b^-/-^*, PKO, P-gp deficient), Bcrp knockout (*Bcrp1^-/-^*, BKO, Bcrp deficient) and triple knockout (*Mdr1a/b^-/-^ Bcrp1^-/-^*, P-gp and Bcrp deficient) mice (Taconic Farms, Germantown, NY), balanced for sex, were used in *in vivo* pharmacokinetic studies. All mice were 8-16 week-old non-tumor-bearing adults, and approximately 15 - 35 g. Sprague-Dawley rat pups were used for the *in vivo* pharmacokinetic studies that followed tumor implantation. Female NSG mice (Jackson laboratory, stock #005557), that were 8 weeks old, were used in pharmacodynamic and efficacy studies.

*In vivo pharmacokinetic studies:*

All dosing solutions were prepared on the day of the study. A serial sacrifice (destructive sampling) design was employed for sample collection. In all the pharmacokinetic studies conducted in non-tumor bearing FVB mice, the group size was 4 at each sampling time point (n = 4). At pre-determined time points, mice were euthanized using a carbon dioxide chamber. Blood was collected by cardiac puncture and transferred to sample tubes coated with heparin. The whole brain was isolated from skull and rinsed with ice-cold distilled water, and superficial meninges were removed by blotting with tissue paper. Plasma was separated by centrifugation of blood samples at 3500 rpm and 4°C for 15 minutes. In the spatial brain distribution study conducted in tumor-bearing rat pups, the group size was 9 (n = 9), and the whole brains were immediately flash frozen following collection. All the samples were stored at -80°C, until further analysis. The concentrations of ispinesib in all the samples were analyzed by LC-MS/MS. Assuming a vascular volume of 1.4% in mouse brain ^5^, the concentrations in brain were corrected for residual drug in brain vasculature.

*Bioluminescence imaging:*

The progression of tumor growth was monitored by bioluminescence imaging (BLI) using an IVIS system. Mice that underwent stereotactic injection with GFP-luciferase expressing GBM1A cells were imaged twice a week. Ten minutes prior to imaging, mice were intraperitoneally injected with d-luciferin at 75 mg/kg body weight (3 mL/kg injection volume). Animals were immobilized using 1.5% − 3.0% isoflurane provided by nose cones within the imager and were kept warm using a heated stage set at 37^o^C during the imaging procedure. Following imaging for about 5 minutes, animals were removed from the IVIS chamber and monitored until they fully recovered from anesthesia.

### *LC-MS/MS analysis*

The samples from *in vitro* and *in vivo* studies were analyzed for ispinesib concentrations using a highly specific liquid chromatography tandem mass spectrometry (LC-MS/MS) assay. Whole blood samples were centrifuged for separating plasma. Uniform homogenates of whole brain specimens were obtained by addition of three volumes of 5% bovine serum albumin (BSA) to whole brains followed by homogenizing using a tissue homogenizer (PowerGen 125; Thermo Fisher Scientific, Waltham, MA). The concentrations of ispinesib were analyzed in plasma and brain homogenate samples using this LC-MS/MS assay. A measured aliquot of sample was spiked with 25 ng of dasatinib as the internal standard for analysis of unknowns. Liquid-liquid extraction was achieved by addition of 1-2 volumes of pH 11 buffer and 5–10 volumes of ethyl acetate to the sample tubes. The sample tubes were then shaked vigorously for 5 minutes, and centrifuged at 7500 rpm and 4°C for 5 minutes. After centrifugation, the organic supernatant layer was collected and dried under nitrogen gas, followed by reconstitution of dried powder residue in 100 µL of freshly prepared mobile phase. An AQUITY ultra performance liquid chromatography (UPLC) system (Waters, Milford, MA) with a Phenomenex Synergi 4 µ Polar-RP 80A column (75 x 2 mm; Torrance, CA) was used for the chromatographic analysis of samples. The mobile phase consisted of 1 mM ammonium formate with 0.1% formic acid (A) and acetonitrile with 0.1% formic acid (B), and was supplied at a constant flow rate of 0.5 mL/min. An isocratic method with 55% solvent A and 45% solvent B was used. The column discharge from the UPLC system was monitored by a Micromass Quattro Ultima mass spectrometer (Waters, Milford, MA). The device consisted of an electrospray interface, and was run by a MassLynx (Version 4.1; Waters) software system. An electrospray probe in positive-ionization mode functioning at a spray voltage of 4.62 kV was used in this analytical method. The samples to be analyzed were introduced into the interface through a heated probe with source temperature of 100°C and desolvation temperature of 350°C. The mass-to-charge (m/z) transitions were 517.20 - 246.96 for ispinesib and 488.21 - 400.99 for dasatinib (internal standard). The retention time was 2.48 minutes for ispinesib and 0.61 minutes for dasatinib. The sample runtime for this analytical assay was 4 minutes.

### *Calculations*

The unbound fractions (fu) in plasma and brain homogenate were calculated as stated below ^1^.

$$f_{u,diluted}=\frac{Drug concentration in buffer (receiver)}{Drug concentration in matrix (donor)} (Equation 1)$$

The unbound fraction in brain was estimated from the measured unbound fraction in diluted brain homogenate (f_u_,_diluted_), using the following equation ^1^.

$$f_{u, brain}=\frac{1/D}{(1/{f_{u, diluted}}-1)+1/D} (Equation 2)$$

where D (equal to 4) represents a dilution factor, accounting for the diluted brain homogenate.

The brain-to-plasma ratios (Kp) were calculated using the area under the concentration-time curve (AUC) or the concentrations (C), in plasma and brain.

$$Kp=\frac{{AUC}_{brain}}{{AUC}_{plasma}} (Equation 3)$$

$$Kp=\frac{C_{brain}}{C_{plasma}} (Equation 4)$$

A comparison of relative drug exposure in brains of wild-type and knockout (*Mdr1a/b^-/-^, Bcrp1^-/-^, Mdr1a/b^-/-^ Bcrp1^-/-^*) mice were made using the distribution advantage (DA), expressed as the Kp in the strain under consideration normalized by the K_p_ in wild-type mice.

$$\mathrm{DA}= \frac{K_{p, knockout}}{K_{p, wildtype}} (Equation 5)$$

The unbound partition coefficient (K_p,uu_) was determined using the following equation.

$$K_{p,uu}=\frac{{AUC}_{brain}X f_{u, brain}}{{AUC}_{plasma} X f_{u, plasma}} (Equation 6)$$

*In vitro* potency data was fitted to “log(inhibitor) vs. normalized response - variable slope” equation in GraphPad Prism version 6.04 (GraphPad, La Jolla, CA) to determine half-maximal effective concentration (EC_50_).

### *Pharmacokinetic data analysis*

The pharmacokinetic parameters from the concentration-time profiles in plasma and brain were determined by non-compartmental analysis (NCA) using Phoenix WinNonlin version 6.4 (Certara USA, Inc., Princeton, NJ). The AUC_plasma_ and AUC_brain_ were calculated using the linear trapezoidal method. The standard errors around the mean of AUC was estimated by the sparse sampling module in WinNonlin ^6^.

### *Statistical analysis*

The statistical analysis of datasets from the conducted studies was performed with GraphPad Prism version 6.04 (GraphPad, La Jolla, CA) software tool. The sample sizes used in the experiments were based on prior work and were estimated based on roughly 80% power to identify a difference of approximately 50% between the groups under consideration. For comparisons between two groups, unpaired t-test was employed. One-way analysis of variance (ANOVA) followed by Bonferroni’s multiple comparison test was used for comparisons involving more than 2 groups. Pairwise statistical testing was accomplished by using two tailed t-test. Log rank test was used for statistical analysis of survival data from *in vivo* efficacy study. For all statistical testing, a significance level of p< 0.05 was employed. Data from all investigations are shown as mean ± standard deviation (S.D.), unless otherwise indicated.

**SUPPLEMENTARY REFERENCES**

1 Kalvass, J. C. & Maurer, T. S. Influence of nonspecific brain and plasma binding on CNS exposure: implications for rational drug discovery. *Biopharm Drug Dispos* **23**, 327-338, doi:10.1002/bdd.325 (2002).

2 Friden, M., Gupta, A., Antonsson, M., Bredberg, U. & Hammarlund-Udenaes, M. In vitro methods for estimating unbound drug concentrations in the brain interstitial and intracellular fluids. *Drug Metab Dispos* **35**, 1711-1719, doi:10.1124/dmd.107.015222 (2007).

3 Gampa, G. *et al.* Brain Distribution of a Novel MEK Inhibitor E6201: Implications in the Treatment of Melanoma Brain Metastases. *Drug Metab Dispos* **46**, 658-666, doi:10.1124/dmd.117.079194 (2018).

4 Gampa, G. *et al.* Brain Distribution and Active Efflux of Three panRAF Inhibitors: Considerations in the Treatment of Melanoma Brain Metastases. *J Pharmacol Exp Ther* **368**, 446-461, doi:10.1124/jpet.118.253708 (2019).

5 Dai, H., Marbach, P., Lemaire, M., Hayes, M. & Elmquist, W. F. Distribution of STI-571 to the brain is limited by P-glycoprotein-mediated efflux. *J Pharmacol Exp Ther* **304**, 1085-1092, doi:10.1124/jpet.102.045260 (2003).

6 Nedelman, J. R. & Jia, X. An extension of Satterthwaite's approximation applied to pharmacokinetics. *J Biopharm Stat* **8**, 317-328, doi:10.1080/10543409808835241 (1998).

**SUPPLEMENTARY FIGURE LEGENDS**

**Figure S1: Chemical structure of ispinesib.**

**Figure S2: Schematic illustration of the methodology for determination of regional drug distribution.** Neonatal Sprague-Dawley rats with TdTomato labelled GBMs were randomized to receive a single dose of 10 mg/kg ip ispinesib with or without simultaneous co-administration of 10 mg/kg ip elacridar on day 21 following intracranial injections. The tumor-bearing brain samples were harvested at 2 hours post dose (n = 9). Coronal sections that were 1-2 mm thick were obtained using an acrylic brain matrix, and a fluorescence microscopy guided punch biopsy method was utilized for the isolation of tumor core, tumor rim (brain adjacent to tumor, BAT) and normal (non-tumor) brain regions. The tumor core was identified as the tumor region with a fluorescence signal that was 5-fold or higher relative to the background signal. The tumor rim was defined as the region around the tumor core with a fluorescence signal 3 to 5-fold higher relative to the background signal. The brain tissue with a fluorescence signal similar to or lower than the background fluorescence was collected as the normal brain. The samples collected from each brain were pooled together, and ispinesib concentrations were determined using LC-MS/MS. The images in the figure are for illustration of the technique, and include images from multiple experiments involving mice and rats.

**Figure S3: Representative fluorescence microscopy images of tumor bearing brain slices.** A fluorescence microscope was used to visualize TdTomato labelled tumors in brain specimens from ispinesib regional brain distribution study. The representative monochromatic images are shown: (A) GBM tumor on a rat brain slice, and (B) tumor core and tumor rim tissues isolated from A.

**SUPPLEMENTARY FIGURES**

**Figure S1**





**Figure S2**


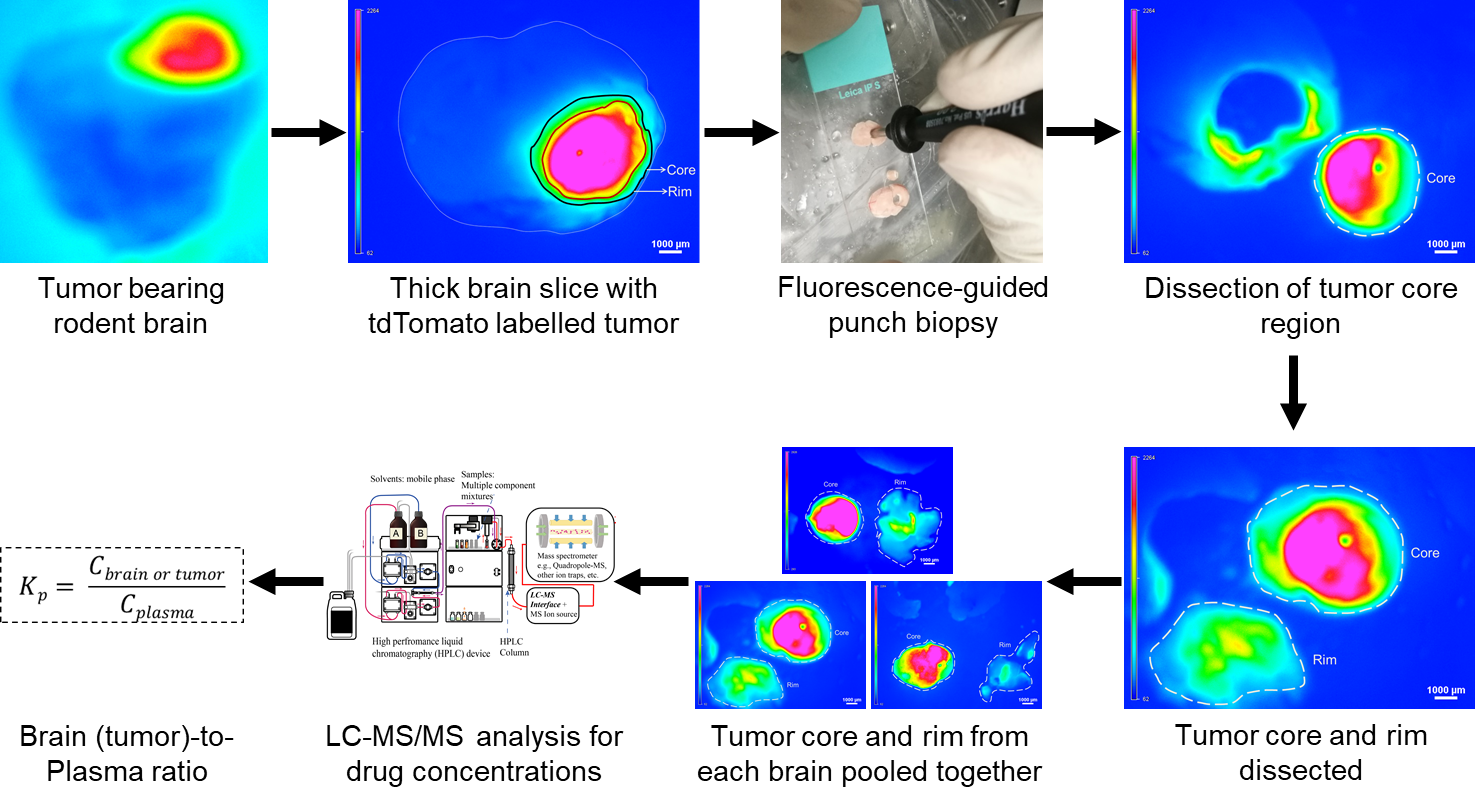


**Figure S3**

**
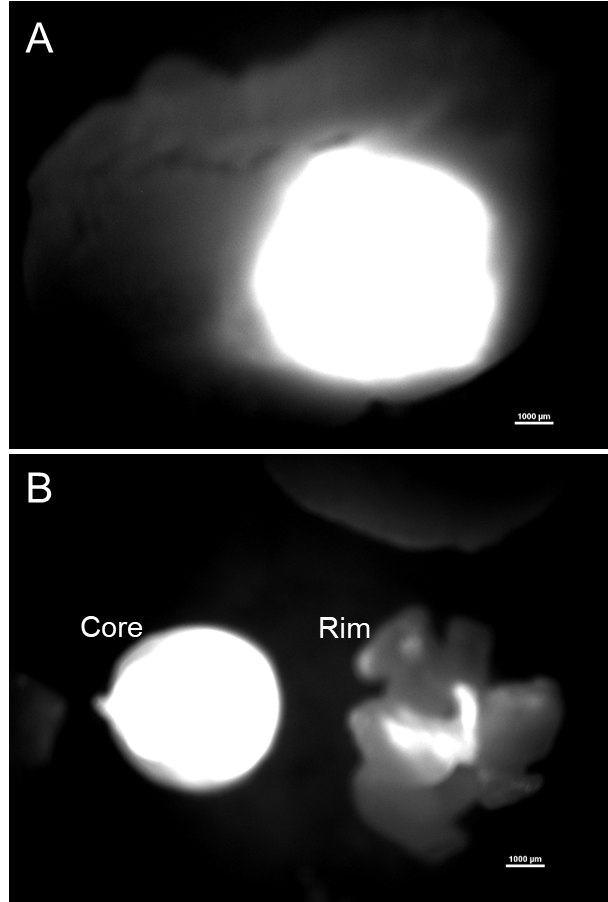
**

**SUPPLEMENTARY TABLES**

**Table S1.** Brain distribution in FVB wild-type, *Bcrp1^-/-^*, *Mdr1a/b^-/-^* and *Mdr1a/b^-/-^ Bcrp1^-/-^* mice following a single dose of 10 mg/kg i.p. ispinesib. Data are presented as mean or mean ± S.D.

| **Time** | **Strain** | **C_plasma_**  **(µg/mL)** | **C_brain_**  **(µg/mL)** | **Kp brain** | **DA** |
| --- | --- | --- | --- | --- | --- |
| 2 hrs | Wild-type | 1.61 ± 0.72 | 0.18 ± 0.09 | 0.11 ± 0.005 | 1 |
|  | Bcrp1a/b^-/-^ | 1.74 ± 0.42 | 0.14 ± 0.02 | 0.08 ± 0.02 | 1 |
|  | Mdr1a/b^-/-^ | 1.75 ± 0.73 | 0.58 ± 0.14 | 0.35 ± 0.08 | 3 |
|  | Mdr1a/b^-/-^  Bcrp1a/b^-/-^ | 1.59 ± 1.07 | 3.77 ± 0.67 | 3.07 ± 1.69 | 28 |
| 6 hrs | Wild-type | 0.87 ± 0.54 | 0.14 ± 0.08 | 0.16 ± 0.03 | 1 |
|  | Bcrp1a/b^-/-^ | 1.06 ± 0.40 | 0.15 ± 0.03 | 0.15 ± 0.05 | 1 |
|  | Mdr1a/b^-/-^ | 0.87 ± 0.54 | 1.10 ± 0.10 | 1.52 ± 0.56 | 10 |
|  | Mdr1a/b^-/-^  Bcrp1a/b^-/-^ | 1.03 ± 0.21 | 5.59 ± 2.85 | 5.20 ± 1.94 | 33 |

Kp brain, the ratio of C_brain_ to C_plasma_ using total drug concentrations

DA (Distribution advantage), the ratio of Kp to Kp_wild–type_

**Table S2.** Brain distribution of ispinesib in FVB wild-type mice following administration of 10 mg/kg i.p. ispinesib with or without 10 mg/kg i.p. elacridar co-administration. Data are presented as mean or mean ± S.D.

| **Time** | **Treatment** | **C_plasma_**  **(µg/mL)** | **C_brain_**  **(µg/mL)** | **Kp brain** | **Fold increase**  **in Kp** |
| --- | --- | --- | --- | --- | --- |
| 2 hrs | Ispinesib | 1.11 ± 0.57 | 0.08 ± 0.02 | 0.10 ± 0.08 | 1 |
|  | Ispinesib  + elacridar | 0.72 ± 0.39 | 0.51 ± 0.36 | 0.80 ± 0.37 | 8 |
| 6 hrs | Ispinesib | 0.73 ± 0.11 | 0.27 ± 0.09 | 0.38 ± 0.14 | 1 |
|  | Ispinesib  + elacridar | 0.79 ± 0.43 | 2.27 ± 0.77 | 3.66 ± 2.64 | 10 |

Kp brain, the ratio of C_brain_ to C_plasma_ using total drug concentrations
